# A203 PERORAL ENDOSCOPIC MYOTOMY (POEM) FOR COMPLEX ACHALASIA AND THE POEM DIFFICULTY SCORE: AN UPDATE

**DOI:** 10.1093/jcag/gwad061.203

**Published:** 2024-02-14

**Authors:** C Ching Hui Yee, M Youssef, M Woo, R Bechara

**Affiliations:** McGill University Faculty of Medicine and Health Sciences, Montreal, QC, Canada; Internal Medicine, University of Toronto, Toronto, ON, Canada; University of Calgary Cumming School of Medicine, Calgary, AB, Canada; Kingston Health Sciences Centre, Kingston, ON, Canada

## Abstract

**Background:**

POEM for achalasia can be challenging in patients with complex achalasia (CA) (i.e., type III achalasia, multiple prior treatments, prior myotomy, and sigmoid type). The POEM difficulty score (PDS) identifies factors that contribute to challenging POEM procedures. (Figure 1) Here, we present an update on the PDS in a series of patients with non-CA and CAs.

**Aims:**

Our aim was to determine whether, with the introduction of the TT-J knife with waterjet functionality, the PDS still maintains a strong correlation with procedural efficiency and technical difficulty.

**Methods:**

We retrospectively reviewed patients who underwent POEM for achalasia between May 2018 to July 2023 at the Kingston Health Sciences Center. 139 consecutive POEMs were performed, with 74 CAs. Primary outcomes include correlation of procedural efficiency with the PDS. Secondary outcomes include clinical success at the last followup and adverse events. Demographics and procedural variables were compared with descriptive statistics (mean ± SD; median, min-max range) and the Mann-Whitney U-test. Pre and post-POEM Echkhardt scores were compared using the Wilcoxon signed-rank test.

**Results:**

74 (56.7% male) complex and 65 (55.4% male) non-complex POEM procedures were carried out. The mean age is 56.7 ± 16.5 years and 47.3 ± 20.2 years in CA and non-CA respectively. PDS correlates moderately with procedural efficiency with a correlation coefficient of 0.595 (Spearman’s Pampersand:003C0.001). A subanalysis is available in Table 1.

The mean efficiency for non-CA was 3.3min/cm±1.2. The median PDS for non-CA was 1 (0-5). In comparison, the mean efficiency for CA were 3.3min/cm ± 1.3 (type III), 5.3min/cm ± 2.3 (prior myotomy), 4.0min/cm ± 1.7 (≥4 prior procedures), and 5.2min/cm ± 2.4 (sigmoid type). The median PDS for CA was 3 (0-6) (type III), 4 (0-8) (prior myotomy), 3 (0-8) (≥4 prior procedures), and 3 (0-6) (sigmoid type).

CA and non-CA POEM procedures were completed with 97% and 98% clinical success, respectively.

**Conclusions:**

PDS continues to moderately correlate with procedural efficiency using the TT-J knife. However, the presence of spastic contractions correlated poorly with procedural efficiency. Thus, it may be omitted with the use of the TT-J knife. The order of increasing difficulty of POEM in CA appears to be type III, prior myotomy, sigmoid type, and ≥4 prior procedures. Multi-center prospective studies are needed to validate PDS in different clinical settings.

Table 1: Sub-analysis of individual PDS factors and procedural velocity

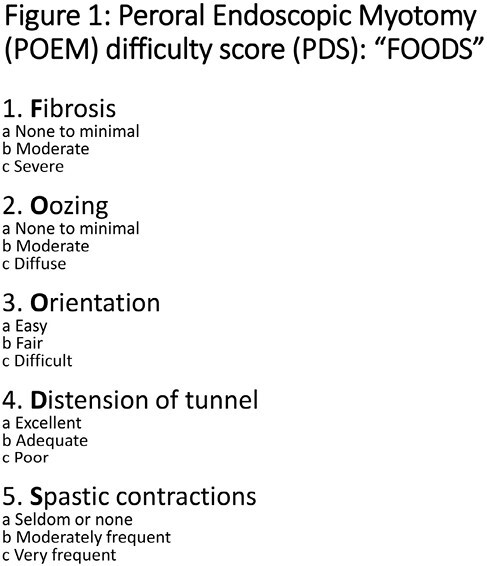

Figure 1: Peroral Endoscopic Myotomy (POEM) difficulty score (PDS): "FOODS"

**Funding Agencies:**

None

**Table:** 

Individual PDS factors	Correlation coefficient (Spearman’s P-value)
Fibrosis	0.520 (Pampersand:003C0.001)
Oozing	0.323 (Pampersand:003C0.001)
Ease of Orientation	0.351 (Pampersand:003C0.001)
Distension of tunnel	0.400 (Pampersand:003C0.001)
Spastic contractions	0.100 (Pampersand:003C0.906)
FOODS factors excluding spastic contractions	0.645 (Pampersand:003C0.001)

